# Long-term down-regulation of GABA decreases orientation selectivity without affecting direction selectivity in mouse primary visual cortex

**DOI:** 10.3389/fncir.2013.00028

**Published:** 2013-03-25

**Authors:** Kenta M. Hagihara, Kenichi Ohki

**Affiliations:** ^1^Department of Molecular Physiology, Graduate School of Medical Sciences, Kyushu UniversityFukuoka, Japan; ^2^CREST, Japan Science and Technology AgencyTokyo, Japan

**Keywords:** primary visual cortex, V1, inhibitory interneuron, orientation selectivity, two-photon imaging

## Abstract

Inhibitory interneurons play important roles in the development of brain functions. In the visual cortex, functional maturation of inhibitory interneurons is essential for ocular dominance plasticity. However, roles of inhibitory interneurons in the development of orientation and direction selectivity, fundamental properties of primary visual cortex, are less understood. We examined orientation and direction selectivity of neurons in GAD67-GFP (Δneo) mice, in which expression of GABA in the brain is decreased in the newborn. We used *in vivo* two-photon calcium imaging to examine visual response of neurons in these mice and found that long-term decrease of GABA led to increase of response amplitude to non-preferred orientation of visual stimuli, which decreased orientation selectivity. In contrast, direction selectivity was not affected. These results suggest that orientation selectivity is decreased in mice with GABA down-regulation during development.

## Introduction

Inhibitory interneurons are important for the development of many brain functions. The functional maturation of inhibitory interneurons plays pivotal roles in activity-dependent ocular dominance plasticity in the visual cortex (Hensch et al., 1998; Fagiolini and Hensch, 2000; Iwai et al., 2003). However, the role of inhibitory interneurons in development of orientation and direction selectivity is less understood, although the preferred orientation of neurons can be modified in an activity-dependent manner (Kreile et al., 2011; Yoshida et al., 2012) and the tuning of cortical inhibition during development can sharpen the orientation selectivity in excitatory neurons (Li et al., 2012). In contrast, many studies have addressed response properties of inhibitory interneurons themselves and acute roles of inhibitory interneuron in shaping or modulating orientation and direction selectivity. Orientation as well as direction selectivity are decreased while inhibition is blocked with a GABA antagonist in cat visual cortex (Sillito, 1975, 1977, 1979; Hata et al., 1988; Sato et al., 1995, 1996; Katzner et al., 2011). In rodent visual cortex, interneurons have only weak selectivity for orientation and direction (Sohya et al., 2007; Kerlin et al., 2010; Ma et al., 2010, but see Runyan et al., 2010) and it is suggested that this broad inhibition sharpens selectivity (Worgotter and Koch, 1991; Liu et al., 2011). Recent studies investigated the acute roles of interneuron subtypes by perturbing each subtype of interneurons with optogenetics or genetics, although results are controversial (Atallah et al., 2012; Lee et al., 2012; Mao et al., 2012; Wilson et al., 2012). Two studies (Atallah et al., 2012; Wilson et al., 2012) suggested that parvalbumin (PV) positive interneurons control response gain and slightly change the visual selectivity of excitatory neurons. Wilson et al. (2012) also suggested that somatostatin (SOM) positive interneurons sharpen visual selectivity. In Dlx1^−/−^ mice that have a selective reduction in cortical dendrite-targeting interneurons (calretinin^+^, neuropeptide Y^+^, or SOM^+^) and a selective increase in PV positive inhibitory interneuron in layer 2/3, neurons showed lower orientation selectivity (Mao et al., 2012). In contrast, another study (Lee et al., 2012) suggested that PV positive interneurons markedly sharpen visual selectivity and that SOM and vasointestinal peptide positive interneurons have no significant effect. Despite of these inconsistencies, these previous works agreed that effects of inhibitory interneurons on the direction selectivity are similar to those on orientation selectivity when groups of interneurons are manipulated.

In this study, we investigated the effects of long-term decrease of GABA. To address this issue, we examined orientation and direction selectivity of neurons in GAD67-GFP knock-in mice, in which expression of GABA in the brain is decreased to 61% of that of wild-type C57BL/6 (Wild-type, WT) mice in the newborn, and 84% in the adult (Tamamaki et al., 2003). A decrease of the amount of GAD67 protein in the brain (Wang et al., 2009) and the impairment of synapse elimination (Nakayama et al., 2012) were also reported in these mice. We found that long-term decrease of GABA led to increase of response amplitude to non-preferred orientation of visual stimuli, and decrease of orientation selectivity, but had no effect on direction selectivity, which suggests that roles of GABA during development is different from those in acute.

## Results

### Visual response of neurons in GAD67-GFP mice

**Figure 1:**
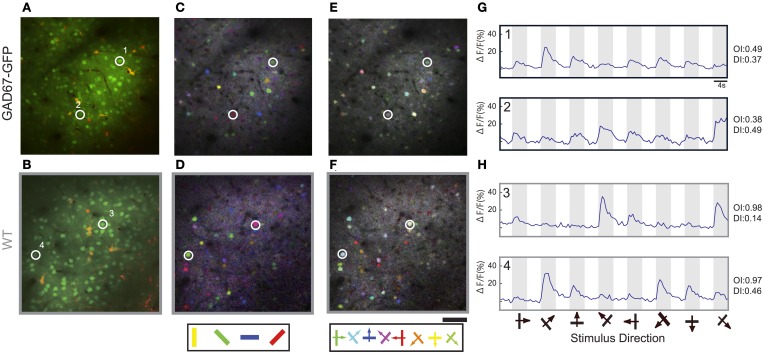
***In vivo* two-photon Ca^2+^ imaging of orientation selectivity and direction selectivity in GAD67-GFP and WT mice. (A,B)** Optical sections of GAD67-GFP **(A)** and WT **(B)** mice at the level of layer 2/3 (about 200 μm depth) in primary visual cortex stained with OGB-1 AM (green) and astrocyte marker Sulfarhodamine 101 (red). **(C,D)** Pixel-based orientation maps of GAD67-GFP **(C)** and WT **(D)** mice, coding for preferred orientation (hue), response amplitude (lightness), and tuning width (saturation). **(E,F)** Pixel-based direction maps of GAD67-GFP **(E)** and WT **(F)** mice, coding for preferred direction (hue), response amplitude (lightness), and tuning width (saturation). **(G,H)** Averaged Δ*F*/*F* time courses of 4 representative neurons, 2 neurons in GAD67-GFP mice **(G)** and 2 neurons in WT mice **(H)**, during stimulus presentation; stimulus orientation and direction are indicated by symbols below. The cells displayed in panels **(A–H)** are labeled by numbers. Scale bars: 50 μm.

To investigate the effect of long-term GABA down-regulation on the visual response of neurons in primary visual cortex, we performed *in vivo* two-photon calcium imaging in GAD67-GFP mice and WT mice. Layer 2/3 neurons in an ~500 μm diameter sphere of mouse visual cortex were bulk loaded with a calcium indicator Oregon Green BAPTA-1 AM (OGB-1) and the astrocyte marker Sulforhodamine 101 (SR101) by pressure injection (Figures 1A,B; Stosiek et al., 2003; Ohki et al., 2005). Responses of OGB-1-labeled cells to moving gratings in eight directions (4 orientations) were studied. The orientation maps [Figures 1C (GAD67-GFP) and D (WT)] and the direction maps [Figures 1E (GAD67-GFP) and F (WT)] obtained from pixel-based analysis (see Materials and Methods) appeared qualitatively similar between GAD67-GFP and WT mice and the salt-and-pepper organization (Ohki et al., 2005; Ohki and Reid, 2007) was evident in both animals. Figures 1G (GAD67-GFP) and H (WT) show the time course of Ca^2+^ signals of neurons, assessed by summing pixel values of OGB-1 fluorescence within the areas regarded as soma (see Materials and Methods). The response magnitude to each of the eight stimulus patterns was obtained from the averaged value during the stimulus presentation. We analyzed changes in fluorescence intensity of 1770 neurons in 5 GAD67-GFP mice and 3718 neurons in 5 WT mice. 626 neurons (35.4%) in GAD67-GFP mice and 1267 (34.1%) neurons in WT were visually responsive (*p* < 0.01, ANOVA across eight directions and a baseline, and Δ*F*/*F*, ratio change in response to preferred direction >2%). Four neurons displayed in Figures 1G,H were visually responsive. There was no significant difference in the percentage of visually responsive neurons between GAD67-GFP and WT mice (*p* > 0.8, Wilcoxon's rank-sum test, 5 GAD67-GFP vs. 5 WT mice).

### Orientation and direction selectivity of neurons in GAD67-GFP mice

**Figure 2:**
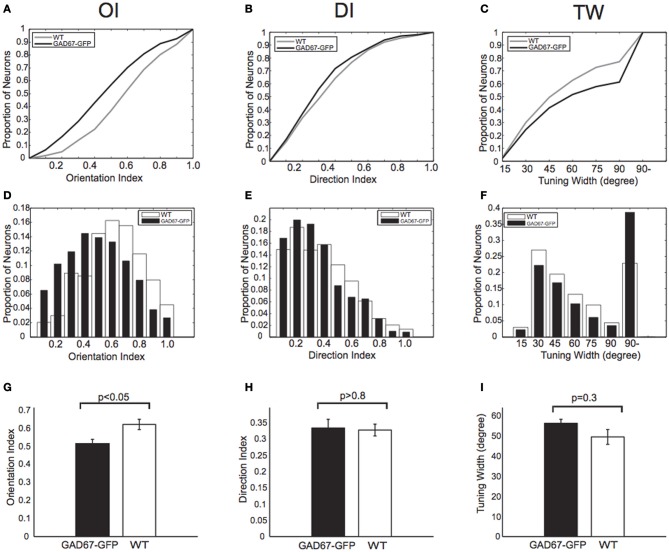
**Orientation and direction indices and tuning width. (A–C)** Cumulative distributions of Orientation Index (OI) **(A)**, Direction Index (DI) **(B)**, and orientation Tuning Width (TW) **(C)** of all neurons responding to drifting gratings in both GAD67-GFP mice and WT mice (*n* = 626 and 1267 neurons, respectively). *p*-values indicate statistical significance in Wilcoxon's rank-sum test, 626 neurons in GAD67-GFP mice vs. 1267 neurons in WT mice. **(D–F)** Distributions of OI **(D)**, DI **(E)**, and TW **(F)**. Same data sets as in **(A)**, **(B)**, and **(C)**, respectively. **(G–I)**: OI **(G)**, DI **(H)**, and TW **(I)** of all visually responsive neurons in GAD67-GFP mice and WT mice (*n* = 5 and 5 mice, respectively). ^*^p < 0.05, n.s.: not significant (Wilcoxon's rank-sum test, 5 GAD67-GFP vs. 5 WT mice). Error bars indicate standard error of means (S.E.M) (across animals).

Based on the response magnitudes to the eight stimulus patterns, we calculated Orientation Index [OI, see Materials and Methods, Figures 2A (cumulative histogram) and D (conventional histogram)], Direction Index (DI, see Materials and Methods, Figures 2B,E), and orientation Tuning Width (TW, see Materials and Methods, Figures 2C,F) of all visually responsive neurons in GAD67-GFP and WT mice. By pooling all the visually responsive neurons from 5 GAD67-GFP and 5 WT mice, we found that OIs of neurons in GAD67-GFP mice were significantly lower than those in WT mice (*p* < 0.05 Wilcoxon's rank-sum test, 5 GAD67-GFP vs. 5 WT mice; Figure 2G), whereas there was no significant difference in DI (*p* > 0.8 Wilcoxon's rank-sum test, 5 GAD67-GFP vs. 5 WT mice; Figure 2H). When we used Orientation Selectivity Index (OSI, see Materials and Methods) instead of OI, we also found lower OSIs in GAD67-GFP mice, although the difference was not significant (*p* = 0.055 Wilcoxon's rank-sum test, 5 GAD67-GFP vs. 5 WT mice). TW was broader in GAD67-GFP mice than in WT mice, although the difference was not significant (*p* = 0.3 Wilcoxon's rank-sum test, 5 GAD67-GFP vs. 5 WT mice; Figure 2I). In a few experiments (not shown), we recorded GFP signals separately from OGB-1 signals, and found the tendency that GFP positive neurons show lower orientation selectivity (OI) than GFP negative neurons in the same mice, as reported by Sohya et al. (2007), although the difference was not significant (*p* < 0.1).

**Figure 3:**
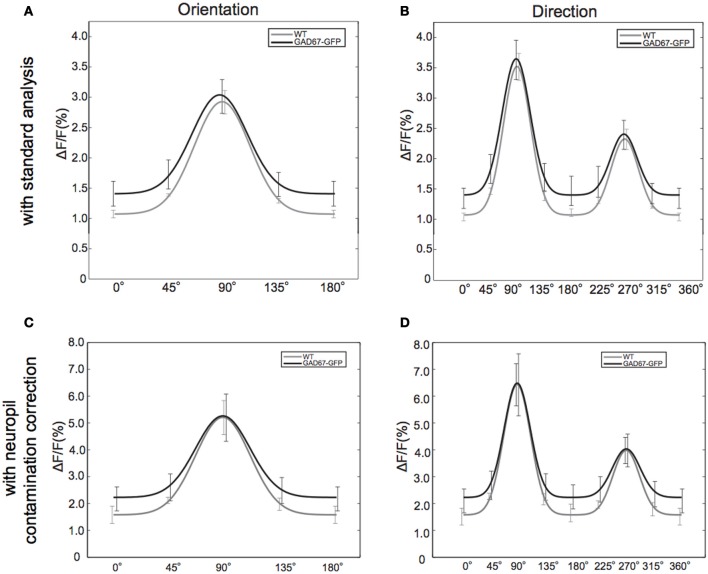
**Tuning curves of the response to each orientation and direction. (A,B)** Orientation **(A)** and direction **(B)** tuning curves are plotted after sifting preferred orientation or direction to 90°, averaging response magnitude and fitting with a Gaussian function (see Materials and Methods). All visually responsive neurons are used. Error bars indicate standard error of mean (S.E.M) (across animals). **(C,D)** Orientation **(C)** and direction **(D)** tuning curves obtained after correcting neuropil contamination (see Materials and Methods).

To understand how OI was decreased but DI was not affected in GAD67-GFP mice, we plotted orientation (Figure 3A) and direction (Figure 3B) tuning curves averaged across all the visually responsive neurons, after shifting preferred orientations (Figure 3A) and directions (Figure 3B) of individual neurons to 90° (see Materials and Methods). We found that the magnitudes of visual responses of neurons in GAD67-GFP mice were larger than those in WT mice (*p* < 0.05, Two-Way ANOVA across orientations or directions and strains, main effect for strains). The elevated visual response was particularly evident in the response to the orthogonal orientation. In contrast, the difference was much smaller in the response to the preferred direction (orientation) and the null direction (Figure 3). Because orientation selectivity is evaluated as the response ratio between the preferred orientation and the orthogonal orientation, this result explains how orientation selectivity was decreased in GAD-GFP mice compared with WT mice.

### Effect of neuropil contamination

We confirmed that neuropil signal contamination does not affect the results shown above, by analyzing the same data set after removing neuropil contamination (see Materials and Methods). After removing neuropil contamination, we found larger Δ*F*/*F* without affecting shapes of tuning curves (Figures 3C,D), suggesting that neuropil contamination decreases Δ*F*/*F* in general but does not affect the tuning curves. Indeed, we obtained consistent results about tuning indexes after correcting neuropil contamination. OIs of neurons in GAD67-GFP mice were significantly lower than those in WT mice (*p* < 0.05) whereas there was no significant difference in DI (*p* > 0.8). TW was higher in GAD67-GFP mice than in WT mice although the difference was not significant (*p* = 0.4).

### Non-linearity of calcium imaging

**Figure 4:**
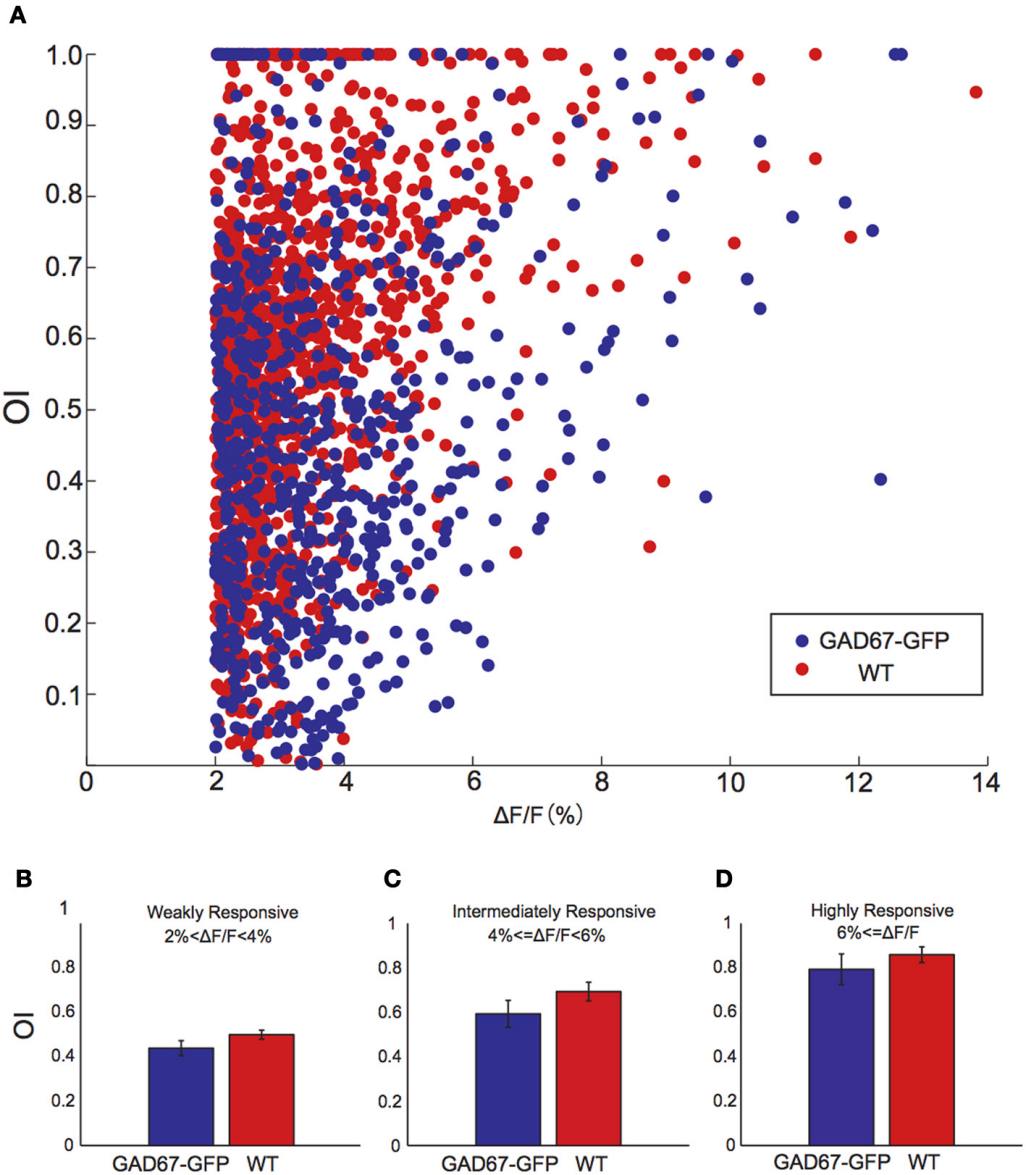
**The relationship between response amplitude and orientation selectivity. (A)** Scatter plot of the relationship between response amplitude and orientation selectivity for all responsive neurons from GAD-67GFP mice (blue) and WT mice (red). **(B–D)** OIs of weakly **(B)**, intermediately **(C)**, and highly **(D)** responsive neurons in GAD67-GFP mice and WT mice (*n* = 5 and 5 mice, respectively). Error bars indicate S.E.M (across animals).

Fluorescence change of calcium indicator is not completely linearly related with firing rate of action potentials, but saturated at high firing rate (Hendel et al., 2008; Nauhaus et al., 2012). Thus, smaller difference of fluorescence change at preferred orientation and direction (Figure 3) could be affected by this non-linearity, although there are many claims that it can be regarded as linear because firing rates of neurons are not high enough to cause the fluorescence saturation in the mouse visual cortex (Niell and Stryker, 2008; Kerlin et al., 2010; Hofer et al., 2011). To test this possibility, we plotted relationship between response amplitude to the preferred direction and OI of each responsive neuron (Figure 4A). The tendency of smaller OI in GAD-GFP67 mice was evident irrespective of response amplitude. We also compared OI of weakly (Δ*F*/*F* < 4%, Figure 4B), intermediately (4% ≤ Δ*F*/*F* < 6%, Figure 4C), or highly (6% ≤ Δ*F*/*F*, Figure 4D) responsive neurons in GAD67-GFP mice with these in WT mice, and confirmed that OI of GAD67-GFP mice were smaller than that of WT mice in all ranges of response amplitude. These results indicate that the decrease of OI in GAD67-GFP mice was not due to the non-linearity of fluorescence change of calcium indicator.

### Effect of contamination of GFP fluorescence in calcium imaging

**Figure 5:**
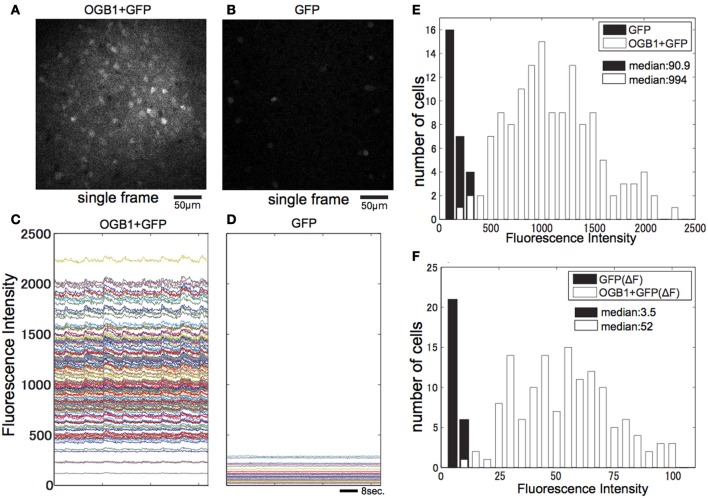
**Effect of the GFP signal in GAD67-GFP mice. (A)** Single-image frame obtained from GAD67-GFP mouse loaded with OGB-1. **(B)** Single-image frame obtained from GAD67-GFP mouse in the same condition as **(A)**. **(C)** Averaged time courses of all visually responsive neurons in imaged plane shown in **(A)** while presenting eight direction visual stimulus. **(D)** Averaged time courses of all GFP positive neurons shown in **(B)** while presenting eight direction visual stimulus. **(E)** Histograms of averaged fluorescence intensity of each neuron shown in **(A)** and **(B)**. **(F)** Histograms of max Δ*F* of fluorescence intensity of each neuron shown in **(D)** and Δ*F* in response to preferred direction visual stimulation of each visually responsive neuron shown in **(C)**.

To examine the effect of contamination of GFP fluorescence in interneurons in GAD67-GFP mice, we measured and compared signals of OGB-1 and GFP (Figures 5A,C) in OGB-loaded GAD67-GFP mice with signals of GFP only in non-loaded GAD67-GFP mice (Figures 5B,D) in the same imaging condition. Signal intensity from GFP-positive cells in animals with only GFP (mean: 91, Figure 5E) was less than 10% of that from OGB-1 and GFP-positive cells in animals with GFP and OGB-1 (mean: 994, Figure 5F). Next, we estimated variability of signals from GFP, which was most likely due to movement of the brain. The variability of signals from GFP was evaluated as the maximum value of Δ*F* of GFP signal in response to eight direction visual stimulation. It was almost negligible (median: 3.5; Figure 5F), compared with Δ*F* (median: 52) of signals from OGB-1 and GFP positive cells in response to visual stimulation with optimal direction. Thus, we concluded that fluorescence of GFP in GAD-GFP mice did not critically affect the signal-to-noise ratio of OGB-1 signal. However, the contamination of GFP signal could have reduced response amplitudes without affecting the shapes of tuning curves, because we assessed response magnitudes as Δ*F*/*F* and the baseline (*F*) of calcium signal could have been overestimated due to the contamination of GFP signal. Our result shows that response amplitudes in GAD67-GFP mice are larger than those in WT, and it is unlikely that this contamination of GFP signal changes our conclusions.

### Decay time of calcium signal

**Figure 6:**
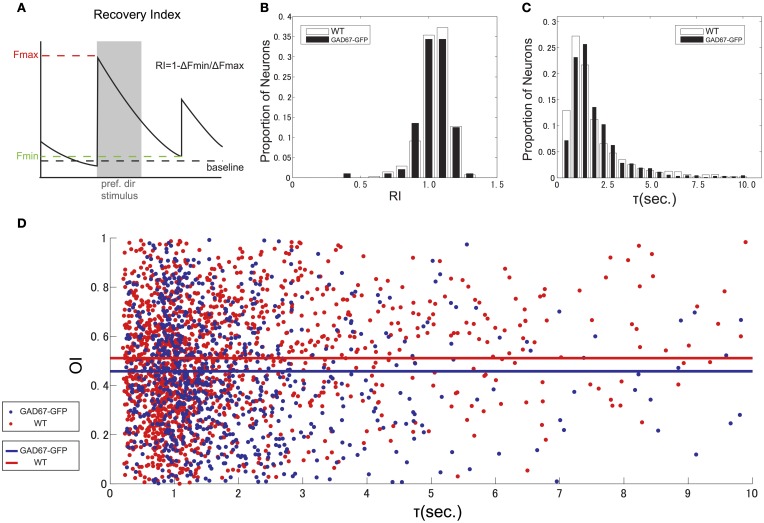
**Decay time of calcium indicator fluorescence. (A)** Scheme indicating how to calculate Recovery Index (RI). **(B)** Distribution of Recovery Index of highly responsive neurons. **(C)** Distribution of decay time constant (τ), calculated by fitting Δ*F* in non-stimulus period with exponential function. **(D)** Scatter plot and regression line of the relationship between tau and orientation selectivity for all responsive neurons from GAD-67GFP mice (blue) and WT mice (red).

To estimate the calcium decay for neurons both in GAD67-GFP and WT mice, we first calculated recovery index (RI) for each cell as 1 – Δ*F*min/Δ*F*max (Δ*F*max: Δ*F* in response to the preferred direction, Δ*F*min: Δ*F* at the last frame of the subsequent no stimulus period, Figure 6A). We estimated RI only for highly responsive (Δ*F*/*F* in response to preferred direction >10%) neurons, because RI could be accurate for highly responsive cells, but inaccurate for weakly responsive cells. RI distributed around 1 (Figure 6B), which suggests that almost all highly responsive neurons recover to baseline before the next stimulus. Next, we calculated decay time constant τ for all responsive neurons (Figure 6C) by fitting Δ*F* in non-stimulus period with an exponential function. Medians of τ were 1.43 s in GAD67-GFP mice and 1.34 s in WT mice, suggesting that 4 s of no stimulation was sufficient to ensure that responses recovered before the next visual stimulus was presented. We found that τ in GAD67-GFP mice was slightly longer than that in WT mice, although the difference was not statistically significant (*p* = 0.35, Wilcoxon's rank-sum test, across cells). To test whether this slight difference of τ affects the estimation of OI, we evaluated the relationship between τ and OI for all visually responsive neurons in both GAD67-GFP mice and WT mice, and found that there is no relationship between τ and OI (correlation coefficient: 0.05 in GAD67-GFP mice, 0.02 in WT mice) in both animals (Figure 6D). This result suggests that slight difference of τ between GAD67-GFP and WT mice did not affect the conclusion.

## Discussion

In summary, this study showed that long-term down-regulation of GABA increased response amplitudes to the non-preferred orientation of visual stimuli and decreased OI without affecting DI, suggesting that GABA down-regulation has a relatively small impact on the development of direction selectivity.

Several previous studies, in contrast, suggested that acute decrease of inhibition affects both orientation and direction selectivity (Sato et al., 1996; Katzner et al., 2011; Atallah et al., 2012). Although it was reported that single-cell activation of SOM affected only orientation selectivity without affecting direction selectivity (Wilson et al., 2012), this is likely because only single-cell was activated. In GAD67-GFP mice, down-regulation of GABA are thought to occur in all inhibitory cortical neurons, and both orientation and direction selectivity would be affected, if the decrease of inhibition were acute. Thus, it is likely that the decrease of orientation selectivity observed in the current study is the effect of GABA decrease in developmental stage.

**Figure 7:**
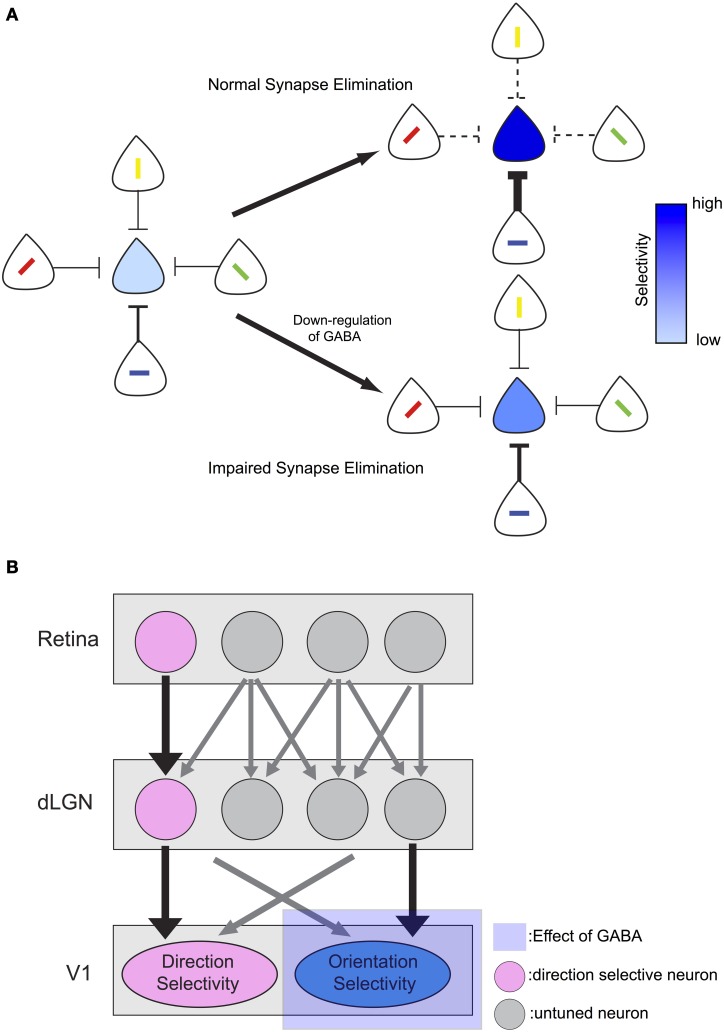
**Models of the effect of GABA *down-regulation* on visual selectivity. (A)** Down-regulation of GABA leads to impaired synapse elimination and decrease orientation selectivity. Depth of blue color of each neuron on the center indicates strength of its selectivity. With normal amount of GABA, selectivity becomes sharp with LTP/LTD mechanism. In contrast, with down-regulation of GABA, selectivity becomes less sharp. **(B)** Input from direction selective retinal ganglion cells (DSGCs) may provide stable direction selective input that is not affected by impaired synapse elimination during development. In contrast, orientation selectivity may be mainly formed from untuned inputs from LGN neurons and depends on normal synapse elimination.

In GAD67-GFP mice, synapse elimination is impaired and the one-to-one connection from excitatory climbing fiber to Purkinje cell is not established normally (Nakayama et al., 2012). Considering that response magnitudes to the non-preferred stimuli were larger in GAD67-GFP mice in the current study, elimination of synapses that convey inputs of non-preferred orientation of visual stimuli may be disturbed by GABA down-regulation during development in GAD67-GFP mice (Figure 7A). We speculate that the down-regulation of GABA during development could result in impaired synapse elimination and decrease of orientation selectivity through the following mechanism. Because of the weak inhibition, postsynaptic neurons in GAD67-GFP mice may respond to their non-preferred stimuli more actively than those in WT mice during development. Then, long-term depression and synaptic elimination may be impaired for synaptic inputs from neurons with different preferred stimuli. This could lead to larger response to non-preferred stimuli even after the developmental period and thus orientation selectivity could be decreased.

Previous studies in mice reported that orientation and direction selectivity develop differently. At the time of eye opening, few neurons have visual response and nearly all of these responsive neurons have both direction and orientation selectivity (Rochefort et al., 2011). As the number of visually responsive neurons increases, neurons with only orientation selectivity appear (Rochefort et al., 2011), suggesting that orientation and direction selectivity mature differently in the developmental stage. On the other hand, the direction selectivity may be unaffected by insufficient inhibition in the developmental stage, possibly because the input from retinal direction selective ganglion cells (DSGCs) may play an important role in generating direction selectivity in mouse visual cortex (Figure 7B). DSGCs project to dorsal lateral geniculate nucleus (dLGN) (Kim et al., 2010; Kay et al., 2011) as well as superior colliculus (Rodieck and Watanabe, 1993; Berson et al., 1998, 1999; Isayama et al., 2000) in mice, and there are direction selective neurons in mouse dLGN (Marshel et al., 2012). Furthermore, at the point of the eye opening, the bias of preferred direction of neurons in the mouse primary visual cortex is similar to that of DSGCs (Rochefort et al., 2011), suggesting that direction selectivity of DSGCs reaches the primary visual cortex and plays a role in the formation of direction selectivity.

A previous study reported that chronic benzodiazepines application during the critical period changes direction selectivity in the kitten visual cortex (Hensch and Stryker, 2004). We speculate the difference between this report and the current result is due to the used species of animals. The percentage of DSGCs in higher mammals is much smaller than that in mice (Cleland and Levick, 1974). Direction selective input from DSGC to cortical neurons through dLGN may be weaker in ferrets and direction selectivity in the visual cortex may be more dependent on GABAergic cortical circuits. Furthermore, in higher mammals such as ferrets or cats, visual cortex has functional columns and inhibitory interneurons have sharp orientation and direction selectivity (Azouz et al., 1997; Cardin et al., 2007), and roles of inhibitory interneurons in development of visual selectivity in higher mammals may be fundamentally different from those in mice.

Decrease of GABA in postnatal development could affect spatial frequency, surround suppression, and receptive field organization, which were not examined in this study. However, from the following reason, we think it is unlikely that differences in orientation selectivity is just epiphenomenon of these changes. If receptive field organization, spatial frequency tuning in GAD67-GFP were changed from those in WT, and if they were the cause of weaker orientation selectivity in GAD67-GFP, the response amplitudes of neurons in GAD67-GFP would be smaller. However, as shown in Figure 3, we found the opposite: response amplitudes to non-preferred orientation became larger in GAD67-GFP animals. It was reported that surround suppression sharpens orientation selectivity (Okamoto et al., 2009). If surround suppression were weakened in GAD67-GFP mice, response amplitudes to preferred orientation in GAD67-GFP mice would be larger than those in WT mice. However, as shown in Figure 3, we did not see the difference in response amplitudes to preferred orientation between GAD67-GFP mice and WT mice. Although it is unlikely that differences in orientation selectivity is just epiphenomenon of the changes in these properties, they could be different in GAD67-GFP mice from those of WT, which should be examined in future studies.

In the current study, we could not completely exclude the possibility of acute effects of decreased GABA and developmental effect of it in retinal circuits, or retino-geniculate projections, because we could not precisely control the amount, period, and area of decreased GABA. However, if the effects were due to changes in retinal circuits or retino-geniculate projections, the effects on direction selectivity would be stronger than those on orientation selectivity, because cells in retina or LGN are more selective to direction than orientation (Elstrott et al., 2008; Marshel et al., 2012). In future studies, stage-specific, subtype-specific, area specific genetic suppression of GABAergic activity will be able to reveal the role of inhibition as well as the roles of each inhibitory interneuron subtype in maturation of the brain circuit at the developmental stage more precisely.

## Materials and methods

All experiments were carried out in accordance with institutional animal welfare guidelines laid down by the Animal Care and Use Committee of Kyushu University, and approved by the Ethical Committee of Kyushu University.

### Transgenic animals

5 GAD67-GFP (Δneo) mice (Tamamaki et al., 2003; postnatal days 60–90) and 5 WT C57BL/6 (postnatal days 60–90; 3 WT litters of GAD67-GFP and 2 genuine WT; we found no difference in response properties between these two WT) were prepared for *in viv*o two-photon imaging.

### Animal preparation and surgery

Mice were anaesthetized with isoflurane (3.0% for induction, 1.5–2.0% during surgery). Incisions were infiltrated with 2% lidocaine gel (xylocaine jelly). A custom-made metal plate was mounted to the skull and a craniotomy was made for calcium imaging. Care was taken to make a craniotomy above the center of monocular region of V1 using stereotaxic coordinate. After surgery, isoflurane concentration was reduced to 0.5–1.0% for visual stimulation and recording experiments.

### Dye loading and *in vivo* two-photon imaging

A total of 0.8 mM Oregon Green 488 BAPTA-1 AM (OGB-1 AM) was dissolved in DMSO with 20% pluronic acid and mixed in ACSF containing 40 mM Sulforhodamine101 (SR101) (all from Invitrogen). A glass pipette (3–5 μm tip diameter) was filled with this solution and inserted into the cortex to a depth of around 250 μm from the surface. OGB-1 AM and SR101 were pressure-ejected from the pipette (5–10 psi for 3–5 s, 3–10 times). Full loading of the OGB-1 AM dye into cortical somata took 0.5–1 h. After confirming loading, the pipette was withdrawn and the craniotomy was sealed with a glass coverslip. Changes in calcium fluorescence in cortical cells were monitored with a two-photon microscope (Zeiss LSM7MP or Nikon A1MP) equipped with a mode-locked Ti:sapphire laser (MaiTai Deep See, Spectra Physics). Excitation light was focused by 25× Olympus (NA: 1.05) or Nikon PlanApo objectives (NA: 1.10). The average power delivered to the brain was <20 mW, depending on the depth of focus. OGB-1 and SR101 were excited at 920 nm. The emission filters were 517–567 nm or 470–550 nm for OGB-1, and 600–650 nm for SR101. We used 515–567 nm filter for OGB-1 in experiments with one GAD67-GFP mouse and one WT mouse and 470–550 nm filter in the rest. We did not see any difference in response amplitude, OI, and DI between these two filters, in both mouse lines.

### Visual stimulation and image data acquisition

We positioned the center of the stimulus monitor at receptive fields of imaged neurons. Before examining orientation selectivity, we determined the average position of receptive fields of imaged neurons by showing small gratings at seven different positions: top-right, top-center, top-left, bottom-right, bottom-center, bottom-left, and center of the monitor, and set the center of the monitor to the average position of receptive fields. Drifting square-wave gratings (100% contrast, 2 Hz temporal frequency) were presented on a 19-inch LCD monitor at eight directions of motion in 45° steps. Spatial frequency was set at 0.033 cycles per degree. Each stimulus started with a blank period of uniform grey (4 s) followed by 4 s of visual stimulation. The eight direction stimuli were presented sequentially and repeated 10–20 times. Care was taken to shield the microscope objective and the photomultipliers from stray light. Images were obtained by Zeiss Zen or Nikon NIS Elements software. A square region of cortex (300–423 × 300–423 μm) was imaged at either 256 × 256 or 512 × 512 pixels at 30–500 ms per frame. In all experiments, images were obtained from depths of 130–300 μm (layer2/3). We imaged less neurons in GAD67-GFP mice, because we imaged from a smaller number of imaging planes in GAD67-GFP mice. However, our imaging planes were always from this range in both animals, and there should not be any bias brought by the difference of the numbers of imaging planes and imaged neurons.

### Data analyses

Images were analyzed in Matlab (Mathworks). When the brain was drifting slowly, more than 2 μm (but not more than several micrometers) over >10 min of scanning time, images were realigned by maximizing the correlation between frames. For pixel-based analysis, images were averaged over stimulus repetitions, and spatially smoothed by Gaussian filter. Fluorescence change (Δ*F*) maps were obtained by subtracting images during the blank period from images during which one of eight directions was presented. Orientation and direction maps were computed from these Δ*F* maps. For cell-based analyses, cells were automatically identified through a series of morphological filters that identified the contours of cell bodies based on intensity, size, and shape. Cell outlines were visually inspected and the rare but clear errors were corrected manually. Time courses of individual cells were extracted by summing pixel values within cell contours. Baseline for each trial and each cell was calculated by averaging the values of the last 0.5 s of the blank periods across all eight directions. Occasionally, slow drift of the baseline signal over minutes was removed by a low-cut filter (cut-off, 2–4 min). Visually responsive cells were defined by ANOVA (*p* < 0.01) across blank and eight direction periods and Δ*F*/*F* >2%. For responsive cells, OI was defined as 1 − (response to orthogonal orientation)/(response to preferred orientation), and DI as 1 − (response to null direction)/(response to preferred direction). Preferred orientation and direction were defined as the angle showing maximum response. Responses to the 4 grating orientations and eight directions were fitted with orientation (Figure 3A) and direction (Figure 3B) tuning curves i.e., a sum of a Gaussian and a constant term, or a sum of two Gaussians and a constant term, respectively. Two Gaussians are forced to peak 180° apart in direction tuning curves. TW was measured as the half-width at half-height (HWHH) of the fitted orientation tuning curves (Maldonado et al., 1997; Ohki et al., 2006). In neuropil contamination removing analysis, the corrected fluorescence signal from a cell body was estimated as follows: *F*_cell−corrected_(*t*) = *F*_cell−apparent_(*t*) − *r* × (*F*_cell−surrounding_ (*t*) − mean(*F*_cell−surrounding_)), where *t* is time and *r* is the contamination ratio. The contamination ratio was calculated in each plane, by fitting time courses of small blood vessels with time courses of surrounding neuropil using least square methods, as follows: (*F*_bv_(*t*) − mean (*F*_bv_)) = *R* × (*F*_bv−surround_(*t*) − mean(*F*_bv−surround_)), where bv is blood vessels in imaged plane. We selected 3 or 4 small blood vessels in each plane, and averaged the values of *R* across those vessels. Decay time constants τ for all responsive neurons were calculated by least squares method, fitting 8 points of Δ*F* in non-stimulus period with an exponential function. After curve fitting, data with Residual Sum of Squares >500, τ > 10,000 (s), or τ < 0.2 (s) were removed as fitting error.

### Conflict of interest statement

The authors declare that the research was conducted in the absence of any commercial or financial relationships that could be construed as a potential conflict of interest.
